# On the Black–White Disparity in Prostate Cancer Mortality

**DOI:** 10.1093/jncics/pkab094

**Published:** 2021-12-31

**Authors:** Otis W Brawley, Sean A Fletcher

**Affiliations:** 1 Sidney Kimmel Comprehensive Cancer Center, Johns Hopkins University School of Medicine, Baltimore, MD, USA; 2 Bloomberg School of Public Health, Baltimore, MD, USA; 3 Department of Urology, Brady Urological Institute, Johns Hopkins School of Medicine, Baltimore, MD, USA

In this issue of the Journal, Wang and colleagues (1) demonstrate that Black Americans are less likely to have access to prostate cancer trials. There has been increasing interest in minority inclusion in clinical trials as a way to overcome disparities. It began with the National Institutes of Health Revitalization Act of 1993, which mandated the inclusion of racial minorities in clinical trials such that valid analysis could be done of the differences between the races (2).

The assumption is that disparities exist because research has not involved Black patients. Contrary to popular belief, minority enrollment in National Cancer Institute (NCI)–sponsored cancer treatment trials was and is proportionate to their representation in the cancer population. In the mid- 1990s, about 3% of Black, White, and Hispanic patients with cancer entered NCI trials (3). Enrollment was and still is low in all demographics. Recent studies suggest racial parity with the proportion enrolled (about 5% of cancer patients of each race and ethnicity are enrolled in NCI sponsored trials), and racial parity in willingness to enter a trial when offered (4). The real problem is less than 10% of all cancer patients in the United States are offered the opportunity to participate in a clinical trial (5). Corporate-sponsored cancer trials enroll more patients per year than NCI-sponsored trials. To our knowledge, data on accrual by race is not published, and there may be disparities.

One should encourage diversity in clinical trials to increase the generalizability of findings. Generalizability of a clinical trial’s findings depends on how closely the enrolled population parallels the population with the disease and the medical care settings treating them. Attention should be given to race, age, and socioeconomic distribution. Generalizability was the basis for the NCI creating the Community Clinical Oncology Program in the 1980s. Even still, those accrued to NCI clinical trials have largely come from suburban America and are more likely to be insured (6). Insurance issues especially hinder access to trials in states that have not expanded Medicaid under the Affordable Care Act. Most of these states are in the south where access to trials is worse.

Racial accrual to prostate cancer trials is of special interest because Black men have a higher risk of diagnosis and, within each stage, a mortality rate more than 1.5 times that of White men. Is this because the treatments are less effective in Black men? The data suggest not.

In observational studies of prostate cancer patients, equal treatment yields equal outcome among Black and White patients treated in the equal-access facilities. The first study to demonstrate this was published in the mid 1990s (7). Over the past two and a half decades, there has been sufficient accrual of patients from racial minority groups to cancer cooperative group prostate cancer trials such that meta-analysis shows that equal treatment yields equal outcome. This has been demonstrated for treatment of localized disease (including active monitoring) and locally advanced and metastatic disease (8-11).

In the National Cancer Database, Black men with high-grade, localized disease treated with radical prostatectomy have a 51% higher mortality compared with White men (hazard ratio [HR] = 1.5, 95% confidence interval [CI] = 1.47 to 1.66) (12). Adjusting for education, median household income, and insurance status, the disparities decreased to 30% (HR = 1.30, 95% CI = 1.26 to 1.34; *P* < 1.00 x 10^-12^). Adjusting for nonclinical factors and comorbidities, the disparity was reduced to 19% (HR = 1.19, 95% CI = 1.15 to 1.23; *P* < 1.00 x 10^-12^). Wang and colleagues (1) importantly and appropriately note that the high association between race and socioeconomic variables means that the effect of each cannot be fully disentangled.

At times, it seems the push for minority inclusion in clinical trials has overshadowed and delayed addressing real reasons for disparities. A substantial proportion of Americans (minorities and the poor) get less than optimal standard care (13). A substantial proportion of women with localized curable breast cancer do not undergo surgery (14). In prostate cancer, there is documented racial variation in receipt of quality surgery, radiation and hormonal therapy, and chemotherapy (15-17). For prostate cancer and most diseases in which there are disparities, it is not that the treatment does not work in Black patients, it is that Black patients are less likely to get the treatment.

Clinical trials are associated with the provision of quality care. Wang and colleagues (1) show that Black patients have less access to clinical trials. They may also be showing that Black patients have less access to high-quality care. There is evidence that doctors who put 2%-3% of their patients on clinical trials take better care of the remaining 97%-98% (18). Indeed, that justifies encouraging patients to see doctors who participate in clinical trials. One can argue that better doctors seek participation in clinical trials or clinical trials make the doctor better. Indeed, both may be true. It is also widely accepted that when a treatment path is unclear for a specific condition, a clinical trial is very appropriate.

Although physicians who participate in trials are more likely to provide high-quality care, one does worry that there may be resource-limited health-care settings where the institution of clinical trials might worsen the overall quality of care provided. There are safety-net hospitals where resources are already strained, and offering clinical trials will tax the system even more (16). The delay for a computed tomography scan will increase, and the burden in the pharmacy and even pathology will grow.

Diversity of populations in clinical study is important not just for generalizability. As we move toward precision medicine and targeted therapies, it is important that we study the distribution of those targets in all populations and not create the situation the National Institutes of Health Revitalization Act was trying to address. Science needs to worry about diversity in observational studies as well as clinical trials.

There is a quandary of race and socioeconomic statuses. Black Americans are more likely to be poor and therefore more likely to get poor care. It is important to realize that the White population is larger, and even though a smaller proportion suffer from socioeconomic disadvantage, the largest number of Americans to receive less than optimal care is likely White and poor. The problem of disparities in health is more than a problem for the 11% of cancer patients who are Black. The correlation of lower socioeconomic status and poor care may motivate greater interest in overcoming disparities in health.

## Funding

P30 CA006973/CA/NCI National Institutes of Health Department of Health and Human Services/United States, The Johns Hopkins Cancer Center grant. OWB receives funding by Bloomberg Philantrophies.

## Notes

**Role of the funders:** The funders had no input into this editorial.

**Disclosures:** OWB: Consultant: Grail, Genentech/Roche, Agilent, Incyte, PDS Biotech, Lyell Immunopharma. SAF: No conflicts to disclose.

**Author contributions:** Writing, original draft: OWB, SAF. Writing, editing and revision: OWB, SAF.

## Data Availability

No new data were generated or analyzed in support of this editorial.

## References

[1] Wang WSDR , Bennette CS, et al Racial disparities in access to prostate cancer clinical trials: a county-level analysis. J Natl Cancer Inst 2021. 10.1093/jncics/pkab093 PMC8763363 35047752

[2] Freedman LS , Simon R, Foulkes MA, et al Inclusion of women and minorities in clinical trials and the NIH Revitalization Act of 1993–the perspective of NIH clinical trialists. Control Clin Trials. 1995;16(5):277–285. discussion 286-9, 293–309. 8582146 10.1016/0197-2456(95)00048-8

[3] Brawley OW , Tejeda H. Minority inclusion in clinical trials issues and potential strategies. J Natl Cancer Inst Monogr. 1995;(17):55–57. 8573455

[4] Unger JM , Hershman DL, Albain KS, et al Patient income level and cancer clinical trial participation. J Clin Oncol. 2013;31(5):536–542. 23295802 10.1200/JCO.2012.45.4553 PMC3565180

[5] Brundage MD. Revisiting barriers to clinical trials accrual. J Natl Cancer Inst. 2021;113(3):219–220. 33022708 10.1093/jnci/djaa156 PMC7936047

[6] Sateren WB , Trimble EL, Abrams J, et al How sociodemographics, presence of oncology specialists, and hospital cancer programs affect accrual to cancer treatment trials. J Clin Oncol. 2002;20(8):2109–2117. 11956272 10.1200/JCO.2002.08.056

[7] Optenberg SA , Thompson IM, Friedrichs P, et al Race, treatment, and long-term survival from prostate cancer in an equal-access medical care delivery system. JAMA. 1995;274(20):1599–1605. 7474244

[8] Odom BD , Mir MC, Hughes S, et al Active surveillance for low-risk prostate cancer in African American men: a multi-institutional experience. Urology. 2014;83(2):364–368. 24286600 10.1016/j.urology.2013.09.038

[9] Dess RT , Hartman HE, Mahal BA, et al Association of Black Race with prostate cancer-specific and other-cause mortality. JAMA Oncol. 2019;5(7):975–983. 31120534 10.1001/jamaoncol.2019.0826 PMC6547116

[10] Spratt DE , Chen YW, Mahal BA, et al Individual patient data analysis of randomized clinical trials: impact of Black race on castration-resistant prostate cancer outcomes. Eur Urol Focus. 2016;2(5):532–539. 28723519 10.1016/j.euf.2016.03.010

[11] George DJ , Halabi S, Heath EI, et al A prospective trial of abiraterone acetate plus prednisone in Black and White men with metastatic castrate-resistant prostate cancer. Cancer. 2021;127(16):2954–2965. 33951180 10.1002/cncr.33589 PMC9527760

[12] Wen W , Luckenbaugh AN, Bayley CE, et al Racial disparities in mortality for patients with prostate cancer after radical prostatectomy. Cancer. 2021;127(9):1517–1528. 32895938 10.1002/cncr.33152

[13] Shavers VL , Brown ML. Racial and ethnic disparities in the receipt of cancer treatment. J Natl Cancer Inst. 2002;94(5):334–357. 11880473 10.1093/jnci/94.5.334

[14] Lund MJ , Brawley OP, Ward KC, et al Parity and disparity in first course treatment of invasive breast cancer. Breast Cancer Res Treat. 2008;109(3):545–557. 17659438 10.1007/s10549-007-9675-8

[15] Lee DJ , Zhao Z, Huang LC, et al Racial variation in receipt of quality radiation therapy for prostate cancer. Cancer Causes Control. 2018;29(10):895–899. 30099628 10.1007/s10552-018-1065-5

[16] Krimphove MJ , Fletcher SA, Cole AP, et al Quality of care in the treatment of localized intermediate and high risk prostate cancer at minority serving hospitals. J Urol. 2019;201(4):735–741. 30414956 10.1016/j.juro.2018.10.024

[17] Wang EH , Yu JB, Abouassally R, et al Disparities in treatment of patients with high-risk prostate cancer: results from a population-based cohort. Urology. 2016;95:88–94. 27318264 10.1016/j.urology.2016.06.010

[18] McFall SL , Warnecke RB, Kaluzny AD, et al Practice setting and physician influences on judgments of colon cancer treatment by community physicians. Health Serv Res. 1996;31(1):5–19. 8617610 PMC1070100

